# Promising Cancer Vaccine for Glioblastoma Therapy: A Focus on mRNA Vaccine

**DOI:** 10.1002/cam4.71187

**Published:** 2025-09-15

**Authors:** Sama Barati, Sahar Ghoflchi, Pejman Hosseinzadeh, Sobhan Pouramini, Hossein Hosseini, Mohammad Jalili‐Nik

**Affiliations:** ^1^ Student Research Committee Mashhad University of Medical Sciences Mashhad Iran; ^2^ Department of Clinical Biochemistry, Faculty of Medicine Mashhad University of Medical Sciences Mashhad Iran; ^3^ Metabolic Syndrome Research Center Mashhad University of Medical Sciences Mashhad Iran; ^4^ Pharmacological Research Center of Medicinal Plants Mashhad University of Medical Sciences Mashhad Iran

**Keywords:** cancer, glioblastoma, mRNA, vaccine

## Abstract

**Background:**

Glioblastoma (GBM) is an aggressive primary brain tumor with poor prognosis and low survival rates. Standard treatments, such as surgery and radiotherapy, are limited by tumor infiltration and resistance. To review current vaccine strategies for GBM, including peptide, virotherapy, cell‐based, and genetic vaccines, with a focus on mRNA vaccines.

**Methods:**

Relevant literature on GBM vaccines and immunotherapy was reviewed to summarize design, mechanisms, and potential clinical applications.

**Results:**

Cancer vaccines aim to activate the immune system to target tumor cells. mRNA vaccines are promising due to their flexibility, rapid production, and strong immune activation, though clinical investigation is ongoing.

**Conclusion:**

Vaccine‐based therapies, particularly mRNA vaccines, hold potential for personalized GBM treatment, but further studies are needed to confirm efficacy and optimize use.

**Abbreviations**
5‐FC: 5‐flucytosine
5‐FU: 5‐fluorouracil
Adv: adenoviruses
ANXA5: Annexin A5
ARPC1B: actin‐related protein 2/3 complex subunit 1B
AV‐GBM: Aivita GBM vaccine
BBB: blood–brain barrier
C FLNC: Filamin
CD: cytosine deaminase
CEA: carcinoembryonic antigen
CGGA: Chinese Glioma Genome Atlas
CMV: human cytomegalovirus
COL1A2: collagen type I alpha 2 chains
CPM: carboxypeptidase M
CSF2RA: colony‐stimulating factor 2 receptor
CTLs: cytotoxic T lymphocytes
CYBA: cytochrome b‐245 light chain
DCs: dendritic cells
DCV: dendritic cell vaccination
EGFR: epidermal growth factor receptor
EGFRvIII: epidermal growth factor receptor III variant
FCGBP: Fc fragment of IgG binding protein
FDA: Food and Drug Administration
FKBP10: FKBP prolyl isomerase 10
GBM: glioblastoma
GMCI: gene‐mediated cytotoxic immunotherapy
GMP: good manufacturing procedures
HLA: human leukocyte antigen
hla‐a: A‐24 alpha chain
hla‐b: B‐41 alpha chain
HSP: heat shock protein
HSV: herpes simplex virus
ICPIs: immune checkpoint inhibitors
IDH: isocitrate dehydrogenase
IFN: interferon
IS: immune subtypes
KDR: Kinase insert domain receptor
KLH: keyhole limpet hemocyanin
LGG: low‐grade glioma
LNPs: lipid nanoparticles
MDSCs: myeloid‐derived suppressor cells
MHC: histocompatibility complex
mOS: median overall survival
mPFS: median progression‐free survival
MSN: moesin
MV: measles virus
MVEdm: MV Edmonton strain
NCI: National Cancer Institute
NeoVax: neoantigen vaccine
nGBM: newly diagnosed glioblastoma
P4HA2: Prolyl 4‐hydroxylase subunit alpha 2
PAMPs: pathogen‐associated molecular patterns
PPV: pneumococcal polysaccharide vaccine
PRRs: pattern recognition receptors
PTEN: phosphatase and tensin
PYGL: glycogen phosphorylase L
RELL1: RELT‐like protein 1
RIG‐I: retinoic acid‐inducible gene I
RRVs: retroviral replicating vectors
SAMD9: sterile alpha motif domain containing 9
SNPs: single nucleotide polymorphisms
SNVs: single nucleotide variants
ssRNA: single‐stranded RNA
TAAs: tumor‐associated antigens
TAMs: tumor‐associated macrophages
TCGA: The Cancer Genome Atlas
TERT: telomerase reverse transcriptase
TICs: tumor‐Initiating Cells
TLR7: toll‐like receptor 7
TME: tumor microenvironments
TMZ: temozolomide
Tregs: regulatory T cells
TSAs: tumor‐specific antigens
WT1: Wilms tumor 1

## 1 Introduction

Glioblastoma (GBM) is an aggressive and highly malignant primary brain tumor, known for its poor prognosis and low overall survival rates despite extensive research and therapeutic advancements [1]. The median overall survival (mOS) for GBM patients with standard treatment is typically around 12–15 months, and the 5‐year survival rate is only about 45%. Survival is often significantly shorter for patients who do not respond well to treatment or experience early recurrence [2, 3].

Current therapeutic strategies have limitations; for instance, surgical resection is the first‐line treatment, but it is rarely curative due to the infiltrative nature of GBM [4]. Post‐surgery, patients typically receive radiotherapy to control local disease progression. However, GBM often recurs, and radiation therapy has limited efficacy over time [5]. Different therapeutic approaches such as photothermal therapy (PTT), Nanomedicines, natural compounds, and targeted therapy also developed for GBM [6, 7, 8, 9, 10]. The oral alkylating agent temozolomide (TMZ) is the standard chemotherapy for GBM and is typically used with radiotherapy [11, 12]. Unfortunately, it has limited effectiveness, and tumors often develop resistance. Recent trials have explored the use of targeted therapies such as targeting vascular endothelial growth factor but have seen limited success due to tumor heterogeneity [13]. GBM is characterized by remarkable heterogeneity in genetic and molecular profile. This complexity contributes to therapy resistance and complicates treatment [14]. Additionally, GBM contains cancer stem cells that are resistant to conventional therapies. These cells can repopulate the tumor after treatment, leading to relapse and poor long‐term outcomes [15]. Also, the Blood–Brain Barrier (BBB) restricts the delivery of systemic therapies to the brain, limiting the effectiveness of many potential anticancer drugs [16, 17]. Even targeted therapies have trouble reaching the tumor cells at sufficient concentrations. Therefore, the limitations in current treatment strategies underscore the need for novel approaches and combination therapies that can effectively target the genetic complexity of GBM, improve drug delivery across the BBB, and overcome tumor resistance.

Cancer vaccines provide a potential strategy in oncology, aiming to stimulate the immune system to recognize and destroy cancer cells [18]. Unlike traditional vaccines that prevent diseases, cancer vaccines are typically therapeutic, designed to treat existing cancer by enhancing the immune response of the body [19]. Cancer vaccines can target specific cancer‐related antigens, allowing the immune system to recognize and attack cancer cells while sparing healthy cells. Also, by training the immune system, cancer vaccines may reduce the likelihood of cancer recurrence by establishing long‐lasting immune memory [20]. Additionally, cancer vaccinations may be integrated with other therapies such as checkpoint inhibitors, to enhance the overall effectiveness of treatment. Moreover, they can be tailored to individual patients, creating a more precise treatment by targeting unique mutations found in a tumor [neoantigens] [21, 22].

mRNA vaccines represent one of the most promising advancements in cancer immunotherapy. Unlike traditional vaccines, mRNA vaccines use synthetic mRNA, which provides instructions to the cells of the body to produce specific proteins [antigens] related to cancer [23, 24]. Once these proteins are expressed on the surface of cells, the immune system can recognize them as foreign and mount an immune response against cancer cells expressing these antigens [25]. The success of mRNA vaccines against COVID‐19 caused interest in using them to target cancer‐specific antigens [26]. mRNA vaccines are still under investigation, but their adaptability, speed of production, and ability to activate a strong immune response make them a powerful new tool in cancer immunotherapy [27, 28, 29]. This paper will review the research conducted on cancer vaccines for GBM by focusing on the mRNA vaccine.

## 2 The Current Designed Vaccine for GBM

**FIGURE 1:**
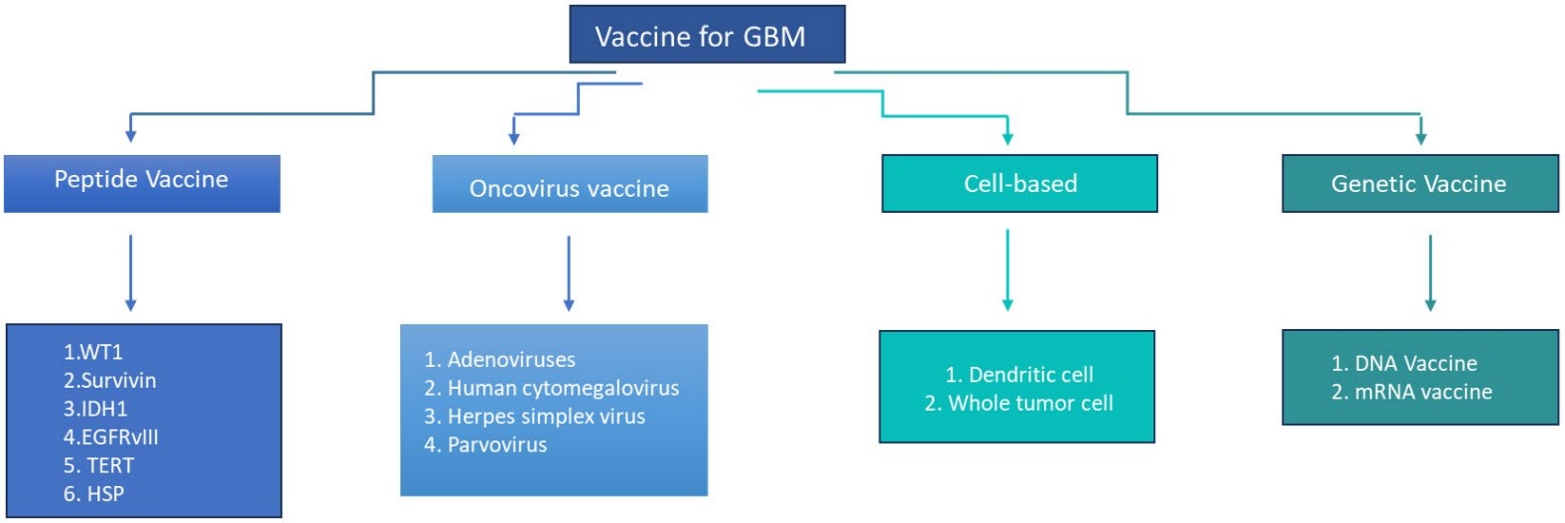
An overview of different vaccines for GBM. EGFRvIII, epidermal growth factor receptor III variant; GBM, glioblastoma; HSP, heat shock protein; IDH1, isocitrate dehydrogenase; TERT, telomerase reverse transcriptase; WT1, Wilms tumor 1.

Designing an effective vaccine for GBM involves careful consideration of the biology of tumors, immune evasion mechanisms, and the unique challenges presented by the central nervous system (CNS) [30, 31]. Recent publications categorize cancer vaccines into four primary types. The following is a brief report on these types. The first type comprises peptide‐based vaccinations that use particular short protein fragments [peptides] targeting cancer cells to activate the immune system against them. The second type is the viral vector vaccine, which utilizes a non‐harmful virus to transport cancer antigens to the immune system. The third type is tumor cell and immune cell vaccines that employ the tumor to educate the immune system to identify cancer cells. The fourth type, nucleic acid‐based vaccinations, applies DNA or RNA to encode cancer antigens and deliver them to the immune system (Figure 1) [32].

### 2.1 Peptide Vaccines

Peptide vaccines usually consist of 8–30 amino acids and target tumor‐specific antigens (TSAs) and tumor‐associated antigens (TAAs) [33, 34]. TSAs are particular to tumor cells and may be patient‐specific. Moreover, TSAs can result from oncolytic viral infections or genetic changes that generate neoantigens. TAAs are more common in tumor cells than TSAs; however, normal cells can express TAAs [35]. Administering TAAs/TSAs via vaccines (e.g., mRNA or dendritic cell‐based) trains the immune system to attack tumors. TSAs potently activate T‐cells and synergize with checkpoint inhibitors (e.g., anti‐PD‐1) to overcome immune evasion, whereas TAAs require cautious selection to avoid autoimmunity [36]. Further, adoptive T‐cell therapies (CAR‐T/TCR‐T) targeting TSAs and adjuvant combinations (e.g., cytokines) amplify cytotoxic responses, collectively enhancing antitumor immunity [37]. For example, WT1 is a well‐known TAA overexpressed in GBM and other malignancies, but it is also detectable at low levels in normal tissues such as kidney podocytes and hematopoietic stem cells [38, 39, 40]. Similarly, survivin, another TAA, is highly expressed in many tumor types but is also present in some normal proliferating cells, including thymocytes and hematopoietic progenitor cells [41, 42]. This expression pattern underlies the potential for autoimmunity and central tolerance, complicating TAA‐targeted vaccine design and necessitating careful antigen selection. Although TSAs and TAAs are thought to trigger autoimmune reactions due to immune cells targeting them, clinical trials have demonstrated that TAAs evoke a more favorable response than TSAs. TSAs are particular to tumors and are derived from genetic changes, unlike TAAs. TSAs targeting needs a customized strategy with low collateral damage risk. They can also provoke strong immune responses and are not susceptible to central tolerance. TSA's cancer vaccines have shown encouraging early results; nevertheless, a meaningful comparison between neoantigen and TAA vaccines is impeded by a lack of clinical data (Table 1) [43].

**TABLE 1:** Active or completed clinical trials for peptide or protein, viral and oncoviral, HSPs, Dendritic cells, and small molecule vaccines in glioblastoma.

Vaccine	Trial title	Phase	*N*	Interventions/Treatment	Status	Results	Clinical trial
WT1	WT2725 in patients with advanced malignancies	1	64	WT1 peptide (WT2725)	Completed	The median OS was 394 days (13.0 months) (95% CI 309–648). The overall immune‐related response rate in solid tumor patients was 7.5% (95% CI 2.6–19.9); response was most prominent in the glioblastoma subgroup.	NCT01621542
	INO‐5401 and INO‐9012 vaccines delivered by Electroporation (EP) and combined with Cemiplimab (REGN2810) in Nd GBM	1/2	52	O6 methylguanine DNA methyltransferase (MGMT) INO‐5401 + INO‐9012 + Cemiplimab + RT + temozolomide (TMZ)	Active, not recruiting	INO‐5401 + INO‐9012, demonstrates acceptable risk/benefit and generates robust systemic immune responses to encoded tumor antigens when administered with cemiplimab and RT/TMZ in newly diagnosed GBM patients. Overall survival is encouraging.	NCT03491683
	Adjuvant dendritic cell‐immunotherapy plus temozolomide in glioblastoma patients (ADDIT‐GLIO)	1/2	20	WT1 mRNA‐loaded DC vaccination +temozolomide	Recruiting		NCT02649582
Survivin	SurVaxM vaccine therapy and temozolomide in treating patients with newly diagnosed glioblastoma	2	66	SVN53‐67/M57‐KLH peptide vaccine + Montanide ISA 51 VG + Sargramostim + TMZ	Active, not recruiting	SurVaxM appeared to be safe and well tolerated. The combination represents a promising therapy for nGBM. For patients with nGBM treated in this manner, PFS may be an acceptable surrogate for OS. Median PFS was 11.4 months and median OS was 25.9 months measured from first dose of SurVaxM.	NCT02455557
	SurVaxM plus adjuvant temozolomide for newly diagnosed glioblastoma (SURVIVE)	2	247	SVN53‐67/M57‐KLH conjugate	Active, not recruiting		NCT05163080
IDH1 EGFRvIII	Phase I trial of IDH1 peptide vaccine in IDH1R132H‐mutated Grade III‐IV Gliomas (NOA‐16)	1	39	IDH1 peptide vaccine targeting the IDH1R132H mutation	Completed	The trial met its primary safety endpoint, with vaccine‐related adverse events restricted to grade 1. Vaccine‐induced immune responses were observed in 93.3% of patients across multiple MHC alleles. Three‐year progression‐free and death‐free rates were 0.63 and 0.84, respectively.	NCT02454634
	IDH1 Peptide Vaccine for Recurrent Grade II Glioma (RESIST)	1	24	PEPIDH1M vaccine in combination with standard chemotherapy (TMZ)	Completed		NCT02193347
	Phase II study of rindopepimut (CDX‐110) in patients With glioblastoma multiforme (ACT III)	2	82	CDX‐110 with GM‐CSF + TMZ	Completed	85% of patients had their Anti‐EGFRvIII antibody titers increase by 4 times or more. At 8.5 months postdiagnosis, the PFS rate was 66% and the 3‐year OS rate was 26%.	NCT00458601
	Phase III study of rindopepimut/GM‐CSF in patients with newly diagnosed glioblastoma (ACT IV)	3	745	Rindopepimut (CDX‐110) with GM‐CSF plus TMZ	Completed	Rindopepimut did not increase survival in patients with newly diagnosed glioblastoma.	NCT01480479
TERT	Anticancer therapeutic by telomerase‐derived universal cancer peptides vaccine in GBM (UCPVax‐Glio)	2	56	UCPVax +TMZ vs. UCPVax	Active, not recruiting		NCT04280848
	INO‐5401 and INO‐9012 vaccines delivered by EP and combined with Cemiplimab (REGN2810) in Nd GBM	1/2	52	MGMT INO‐5401 + INO‐9012 + Cemiplimab + RT + TMZ	Active, Not Recruiting	—	NCT03491683
Cytomegalovirus	DC migration study for newly‐diagnosed GBM (ELEVATE)	2	64	pp65 DC vaccine + TMZ vs. pp65 Dendritic cells vaccine + TMZ + preconditioning	Completed	—	NCT02366728
	Vaccine therapy for the treatment of newly diagnosed glioblastoma (ATTAC‐II)	2	175	CMV pp65‐mRNA loaded Dendritic cells + GMCSF + Td + TMZ	Completed		NCT02465268
	DC migration study to evaluate TReg depletion In GBM patients with and without Varlilumab (DERIVe)	2	43	Human CMV pp65‐LAMP mRNA‐pulsed autologous Dendritic cells + TMZ	Active, not recruiting		NCT03688178
Heat shock protein	HSPPC‐96 vaccine with temozolomide in patients with newly diagnosed GBM (HeatShock)	2	70	HSPPC‐96 + TMZ + Carcinotomy	Completed	Vaccination with autologous tumor‐derived heat shock proteins may improve survival for GBM patients when combined with standard therapy and warrants further study. Median OS for patients with high PD‐L1 expression on myeloid cells was 18.0 months as compared to 44.7 months for patients with low PD‐L1 expression (*p* = 0.007)	NCT00905060
Dendritic Cell Vaccination	Study of a drug [DCVax‐L] to treat newly diagnosed GBM brain cancer (GBM)	3	348	DCVax‐L, Autologous Dendritic Cells Pulsed With Tumor Lysate Antigen	Unknown	Considerable increase in survival when compared to comparable external controls. Median OS in ndGB (from randomization): 19.3 months versus 16.5 months. mOS in rGB (from relapse): 13.2 months versus 7.8 months	NCT00045968
	A study of ICT‐107 immunotherapy in glioblastoma multiforme (GBM)	2	124	ICT‐107	Completed	PFS was extended by 2.2 months, yet no notable enhancement in OS was demonstrated. The HLA‐A2 patient group exhibited greater immune response levels compared to the HLA‐A1 patient group	NCT01280552
	Efficiency of vaccination with lysate‐loaded dendritic cells in patients with newly diagnosed glioblastoma (GlioVax)	2	136	Autologous, tumor lysate‐loaded, mature dendritic cells	Recruiting		NCT03395587
	Dendritic cell immunotherapy against cancer stem cells in glioblastoma patients receiving standard therapy (DEN‐STEM)	2/3	60	Dendritic cell immunization + Adjuvant temozolomide	Recruiting		NCT03548571
	Pembrolizumab and a vaccine (ATL‐DC) for the treatment of surgically accessible recurrent glioblastoma	1	40	Dendritic Cell Tumor Cell Lysate Vaccine + Pembrolizumab + Placebo Administration + Poly ICLC	Recruiting		NCT04201873
Oncoviral vaccines	Study of a retroviral replicating vector combined with a prodrug to treat patients undergoing surgery for a recurrent malignant brain tumor	1	58	DNX‐2440 conditionally replication‐competent adenovirus with O × 40 ligand (T‐cell stimulator)	Completed	Dose‐limiting toxicity	NCT01470794
	PVSRIPO in recurrent malignant glioma	2	62	G207 (modified oncolytic strain of HSV‐1) single‐dose inoculation	Active, not recruiting	OS at 24 months	NCT02986178
	Safety and efficacy of the ONCOlytic VIRus armed for local Chemotherapy, TG6002/5‐FC, in recurrent glioblastoma patients (ONCOVIRAC)	2	78	Ad5‐DNX‐2401 (oncolytic adenovirus) in bone marrow	Unknown status	Progression at 6 months	NCT03294486
	Combination adenovirus + pembrolizumab to trigger immune virus effects (CAPTIVE)	2	49	DNX‐2401+ pembrolizumab	Completed	DNX‐2401 combined with pembrolizumab was well received by certain patients, showing significant survival advantages. (mOS) was 12.5 months; the OS rates at 12 and 18 months were 54.5% and 20.8%, respectively.	NCT02798406
	A study of the treatment of recurrent malignant glioma with rQNestin34.5v.2 (rQNestin)	1	108	PVSRIPO (genetically recombinant nonpathogenic poliovirus: rhinovirus chimera) ± lomustine	Recruiting	MTD	NCT03152318

#### 2.1.1 WT1

Wilms tumor 1 (WT1) is identified as a GBM TAA that is related to several solid tumors and leukemias, as well as promoting carcinogenesis. Immunotherapy against WT1 has been beneficial in treating recurrent GBM, as most GBM specimens overexpress WT1 [44]. The WT1 protein has been designated by the National Cancer Institute (NCI) as the main validated target for the development of cancer vaccines [45, 46]. Peptide vaccines against WT1 have been used in several clinical trials, and the results have shown a reduction in detectable WT1 transcript levels and therapeutic effects [47]. WT1 expression can be seen at low levels in normal cells, but it is increased in certain malignancies, including GBM [48]. This suggests that responses from anti‐WT1 T cells, especially those produced by high‐avidity T cells, could eventually become tolerogenic [49]. Researchers have recently proposed that one way to help leukemia patients overcome immunological tolerance is by the production of low‐avidity CTLs. On the other hand, high‐avidity T cells are more efficient in eliminating CML than low‐avidity TCRs for WT1‐MHC. While highly immunogenic, WT1 poses a challenge because it is also expressed at low levels in healthy tissues (e.g., kidneys, hematopoietic precursors), leading to central tolerance—a process that deletes or dampens high‐affinity T cells reactive to self‐antigens like WT1 [48, 49, 50, 51, 52, 53, 54, 55].

#### 2.1.2 Survivin

Survivin is an apoptosis‐inhibitory protein that is associated with a poor prognosis and a low overall survival rate in CNS malignancies, including gliomas and other tumors [56]. Survivin has the ability to interfere with apoptosis, boost cancer stem cell proliferation, increase tumor cell invasion, and contribute to chemotherapy resistance in cancer cells [57]. Strong tumor antigen survivin binds to MHC class I molecules on cancer cell surfaces to provide T lymphocytes with a stimulatory ligand; that is, these modified peptides also stimulate higher‐avidity T‐cell responses, overcoming preexisting tolerance and decreasing T‐cell exhaustion by enhancing tumor clearance and synergizing with checkpoint inhibitors [58, 59]. Vaccination boosts this process by expanding low‐affinity survivin‐reactive T cells and converting them into potent, durable effectors—effectively transforming modest baseline immunity into robust antitumor activity, and given the modest immunogenicity of wild‐type survivin, the vaccination may strengthen preexisting immunity [60, 61, 62]. Compared to the matching wild‐type survivin peptide, SurVaxM (SVN53–67/M57‐KLH), a recently developed peptide vaccine made from the human survivin protein sequence (containing amino acids 53–67), elicited a stronger antitumor immune response against tumor cells [60]. In a phase IIa trial, the safety, immunologic effects, and survival of newly diagnosed GBM (nGBM) patients receiving adjuvant TMZ plus SurVaxM after surgery and chemoradiation were assessed. SurVaxM was shown to be well tolerated and safe [63]. For nGBM, the combination offers a viable treatment. When treating nGBM patients in this way, progression‐free survival (PFS) could be a suitable overall survival (OS) substitute. This is based on the strong correlation observed between prolonged PFS and improved OS in the study population. Additionally, PFS offers an earlier and less confounded measure of therapeutic efficacy in aggressive cancers like GBM, where OS can be affected by postprogression treatments and extended follow‐up times [63, 64].

#### 2.1.3 IDH1

The metabolic enzymes known as Isocitrate dehydrogenase 1/2 (IDH1/2) are encoded by the IDH1 and IDH2 genes and located on chromosomes 2 and 15, respectively [65]. IDH has been considered a potential TSA since mutation in it mainly occurs in tumor cells, not in normal human cells [66]. Mutations in IDH1, particularly the R132H variant, are found in approximately 80% of low‐grade gliomas (LGG) and are a hallmark of secondary GBM, but are rare in primary GBM. An IDH1 gene mutation suggests that the GBM is a low‐grade secondary glioma [67]. Numerous human malignancies, including gliomas, have been found to have mutations in the genes encoding these enzymes [68]. These mutations cause oncometabolite 2‐hydroxyglutarate (2HG) synthesis, genomic instability, and neoplastic transformation, which are consistently distributed in the catalytically active areas of these enzymes [69]. Schumacher et al. initially attempted to create vaccinations against mutant IDH. Mice were injected with a peptide consisting of twenty amino acids, which covered a portion of the altered catalytic pocket of the IDH enzyme. Mice were injected with a 20‐amino‐acid peptide derived from the mutated catalytic pocket of IDH (e.g., R132H), which drives cancer via D‐2HG production. Antigen‐presenting cells (APCs) internalized and processed the peptide, presenting it on MHC molecules to activate tumor‐specific CD8+/CD4+ T cells. By targeting the mutation‐induced neoepitope—absent in wild‐type IDH—these T cells bypassed immune tolerance and selectively killed IDH‐mutant tumor cells, reducing D‐2HG levels. The peptide's length likely required in vivo trimming to MHC‐compatible epitopes (8–12‐mers), with adjuvants potentially enhancing APC priming. This approach mirrors clinical vaccine strategies against IDH‐mutant gliomas, where mutant‐IDH peptides induce cytotoxic responses without attacking normal tissues. T‐helper cells specific to this mutation demonstrated a robust immunological response, according to the findings [70]. Pellegatta et al. developed an immunologically viable glioma model of the R132H mutation. Their results revealed that peptide vaccination may delay otherwise fatal intracranial glioma by about a month and, in a fraction of cases, cure it [71]. The initial phase 1 trial in humans of the IDH1 peptide vaccine was NOA‐16 (NCT02454634). Patients with grade 3 and 4 IDH1R132H astrocytoma who had just been diagnosed were enrolled [72, 73].

#### 2.1.4 EGFRvIII

A distinct subset of tumor cells (about 33%) expresses the permanently active wild‐type tyrosine kinase mutant known as the epidermal growth factor receptor III variant (EGFRvIII). It is a viable target for the creation of customized immunotherapies due to its level of specificity [74]. Mechanistically, EGFRvIII promotes glioma invasion by activating HIF‐1a in a STAT3‐dependent manner and by overexpressing the antiapoptotic protein Bcl‐xL [75]. EGFRvIII exhibits persistent ligand‐independent activity even in the absence of a functioning ectodomain, which would otherwise confer ligand specificity. In addition to increased carcinogenesis, dysregulated EGFR signaling has been linked to metastasis, resistance to chemotherapy and radiotherapy, and metastasis [76].

This oncogenic protein, EGFRvIII, is a very appealing tumor‐specific target for the development of a GBM vaccination since it promotes proliferation [77]. The shortened extracellular domain produces a unique tumor neoantigen that is specific to GBM cells in both mice and people [78]. This prompted the creation of the peptide‐based vaccination rindopepimut (CDX‐110). Rindopepimut is a peptide vaccination that kills GBM cells that express EGFRvIII by including 14 amino acids from the EGFRvIII fusion site [77, 78, 79]. Three discrete reasons emerged: first, strong immunogenicity; second, low toxicity that is, KLH has an excellent safety profile in humans, with only mild side effects (e.g., transient fever or injection‐site reactions); and third, wide distribution, that is, KLH is commercially available, standardized, and compatible with clinical‐grade manufacturing, facilitating scalable vaccine production of keyhole limpet hemocyanin (KLH). This vaccine is frequently used in conjunction with it. In vivo, KLH stimulates immune responses that are dependent on T and B cells, hence fostering antigenic immune responses [80]. In fact, KLH enhances the immunogenicity of EGFRvIII‐targeted vaccines (e.g., rindopepimut) by acting as a potent carrier protein. As a foreign T‐cell‐dependent antigen, KLH stimulates robust CD4+ T‐cell help, which in turn drives both antibody production against EGFRvIII and cytotoxic CD8+ T‐cell responses to eliminate EGFRvIII‐expressing tumor cells. Its low toxicity, commercial availability, and ability to break immune tolerance make KLH an ideal partner for overcoming the weak intrinsic immunogenicity of tumor‐specific peptides like EGFRvIII. However, while KLH boosts antigen‐specific immunity, its efficacy in clinical settings may still require combination with other therapies to counteract tumor immune evasion mechanisms.

Another study by Sampson et al. explored the mechanisms underlying disease progression after prolonged survival with EGFRvIII‐targeted vaccination. EGFRvIII, a tumor‐specific mutation, is expressed in a subset of GBM and offers a target for vaccine‐based therapies. Patients achieving extended PFS following EGFRvIII peptide vaccination were analyzed for EGFRvIII expression and immune profiles. Tumor biopsies and blood samples were evaluated to identify resistance mechanisms. The outcome illustrated that tumor recurrence was associated with the loss of EGFRvIII expression and immune evasion strategies, such as T‐cell exhaustion. These findings emphasize the need for combinatorial approaches to overcome immune escape [81]. In the same vein, the ACT IV trial investigated the efficacy of rindopepimut, an EGFRvIII‐targeting peptide vaccine, combined with TMZ. This randomized, double‐blind, phase III trial enrolled newly diagnosed EGFRvIII‐expressing GBM patients. Participants received rindopepimut or placebo alongside standard chemoradiotherapy. Outcomes included PFS and OS. The most important clinically relevant finding was the vaccine generated robust immune responses but did not improve OS or PFS compared to the control group [82]. Although the immunotherapeutic targeting of EGFRvIII may effectively eradicate neoplastic cells it might be impeded by concurrent myelosuppressive chemotherapy, such as TMZ, that offers a survival advantage in GBM. A phase II, multicenter study was conducted to evaluate the immunogenicity of an investigational EGFRvIII‐targeted peptide vaccine in patients with GBM receiving therapy with numerous cycles of standard‐dose or dose‐intensified TMZ. The results indicated that EGFRvIII‐specific immune responses emerged in all patients receiving either treatment; however, the DI TMZ regimen elicited a more pronounced amplitude of humoral and delayed‐type hypersensitivity reactions. Vaccination targeting EGFRvIII elicits immune responses in patients despite lymphopenia generated by TMZ therapy and eradicates EGFRvIII‐expressing tumor cells without causing autoimmunity [83, 84, 85, 86].

#### 2.1.5 TERT

The telomerase reverse transcriptase (TERT) protein is an important enzyme complex in eukaryotic species that helps preserve and lengthen telomeres, enhancing the possibility of cell division [87]. Because TERT mutations reactivate the telomerase enzyme and immortalize malignant cells, they have been associated with the development of cancer, especially in the promoter region of the gene [88, 89]. TERT promoter mutations are common in GBM and are linked to varying prognoses based on additional genetic variables. For instance, in GBM with IDH mutations, the presence of a TERT promoter mutation is particularly linked to a better prognosis. Nonetheless, patients with mutations in the TERT promoter and unmethylated MGMT promoters typically have the worst prognoses [90, 91, 92]. To potentially treat TERT‐related tumors, researchers are presently investigating therapeutics that target TERT activity, such as vaccinations and small‐molecule inhibitors [93]. Amen et al. observed that cancer‐cell‐specific TERT inhibition via GABPB1L decrease leads to short‐term growth inhibitory effects and a compromised DNA damage response, which significantly raises the susceptibility of GBM tumors to frontline chemotherapy. Additionally, their findings support the use of TMZ chemotherapy in conjunction with GABPB1L peptide suppression as a potentially effective treatment for GBM [93]. In another study, researchers paired this strategy with the use of survivin‐mRNA and hTERT‐transfected DCs to aid in monitoring induced immunity and may serve as therapeutic targets. Their findings imply that it is possible to establish autologous cancer stem cell cultures under good manufacturing procedures (GMP). It is clear that immunization against cancer stem cells can increase the length of time without recurrence and is safe and well‐tolerated [94, 95, 96, 97, 98].

#### 2.1.6 HSP

A novel vaccination strategy called the heat Shock Protein (HSP) vaccine works by using proteins with molecular chaperone activity to stop biological macromolecules that are impacted by ions, oxygen, and temperature from denaturing [99, 100]. HSPs may be useful in tumor tissues where aberrant proteins are abundant because they can reassemble misfolded proteins and direct the breakdown of aberrant ones. Studies have revealed that HSPs such as HSP96 can trigger potent immune responses and are strongly linked to gliomas. Before brain tumor‐derived HSP96 is internalized and presented as an HSP96‐chaperoned tumor antigen on class I and class II MHC, the HSP96 complex is first attached to CD91 on antigen‐presenting cells (APCs), resulting in robust immunogenicity. The advantage of the HSP vaccine over other tumor vaccines is that it better induces CD4^+^ and CD8^+^ T‐cell immune responses due to its highly precise interaction between HSP96 and APCs [101].

The HSP vaccine has the benefit of being selective in how it interacts with antigen‐presenting cells, which stimulates strong T‐cell immune responses [101]. The vaccination did not cause any major adverse effects, and the most common adverse event was minor injection site erythema. Similar to other studies, the single‐arm phase II trial that followed included 41 patients with recurrent GBM and revealed that the mOS of the HSP96 group was 42.6 weeks following vaccination without experiencing any significant side effects (Table 1) [102, 103, 104].

#### 2.1.7 Personalized Peptide Vaccines

Personalized peptide vaccination (PPV), appropriate peptide antigens for vaccination are screened and selected from a list of vaccine candidates in each patient, based on preexisting host immunity [105]. A phase III trial of PPV for HLA‐A24+ recurrent GBM found that the trial met neither the primary nor secondary endpoints. Unfavorable factors for the mOS of 58 PPV patients compared to 30 placebo patients included SART2‐93 peptide selection, ≥ 70 years old, > 70 kg body weight, and performance status [106]. The mOS for PPV patients without SART2‐93 selection plus one of these three favorable factors was significantly longer than that for the corresponding placebo patients. Preexisting immunity against all 12 warehouse peptides was significantly depressed in patients with SART2‐93 selection compared to those without. Biomarkers correlating for favorable OS included a lower percentage of CD11b + CD14 + HLA‐DR low immunosuppressive monocytes and a higher percentage of CD4 + CD45RA‐activated T cells. Another phase III study utilizing personalized peptide vaccine (PPV) for HLA‐A24+ recurrent GBM did not achieve the main or secondary goals. Detrimental variables for the mOS of 58 patients receiving PPV compared to 30 patients receiving placebo were SART2‐93 peptide selection, age ≥ 70 years, and body weight > 70 kg. The mOS for PPV patients without SART2‐93 selection, along with one of three favorable characteristics, was considerably longer than that of the placebo. Preexisting immunity against all 12 peptides was considerably decreased in individuals with SART2‐93 selection compared to those without. Biomarkers associated with improved mOS included a reduced proportion of CD11b + CD14 + HLA‐DR reduced immunosuppressive monocytes and an elevated proportion of CD4 +CD45RA‐activated T cells. Taken together, the vaccine elicited tumor‐specific immune responses, with prolonged PFS in responders, highlighting its potential in tailored immunotherapy [106, 107, 108, 109].

### 2.2 Virotherapies for Treatment of GBM

#### 2.2.1 Oncolytic Virus's Vaccines

The weak pathogenic (infectious) viruses with genetic modification are made oncolytic viruses, which increase anticancer effects despite ceasing to destroy normal cells. The oncolytic effect results from direct lysis of the cancer cells due to the virus self‐replication in host cancer cells [110, 111]. In recent years, modified viruses have become more widely used, opening the door for their application in oncotherapy [112]. Viruses can activate the immune system by inducing innate responses and enhancing specific responses to tumor antigens, thereby significantly increasing the efficacy of vaccinations [113].

In clinical trials enrolling patients with glioma, Parvoviridae, Picornaviridae, Retroviridae, Paramyxoviridae, Adenoviridae, Reoviridae, and Herpesviridae are the most frequently utilized viruses. However, the most important problem is that most viruses, apart from Parvoviridae, cannot pass the BBB due to their lack of tropism; thus, issues have to be solved in this type of therapy [114]. Additionally, the expansion of the extracellular matrix is linked to a desmoplastic condition, one of the changes tumor microenvironments (TME) endure [115]. Herpesviruses rely on the tumor's surrounding microenvironment for entry, particularly due to the elevated expression of integrins in glioma cells, which facilitates their cellular access. However, the treatment of GBM remains highly challenging because of the brain's immune‐privileged status and the strongly immunosuppressive tumor microenvironment [116]. To overcome these obstacles, research into novel delivery systems that can either penetrate the BBB or inoculate directly into the tumor site is just as important as looking for therapies that stimulate an immune response [117]. The subsequent properties of viruses and the research conducted on them position them as viable candidates for oncolytic viral treatment in GBM.

Adenoviruses (Adv) are non‐enveloped, double‐stranded DNA viruses that have an icosahedral capsid. At least three oncolytic viruses have been developed for the treatment of GBM, making it one of the most often used oncolytic viruses [118]. Herpes simplex virus (HSV)‐1 is a virus containing double DNA strands in its envelope that infects human neural tissues without warning. Therefore, it shows potential as a viable choice for oncolytic virotherapy in GBM. Multiple modified oncolytic HSVs (oHSVs) with varying attenuation levels have been developed for treating GBM, with six of them progressing to clinical trials. HSV‐1 is among the most extensively studied oncolytic viruses (OV) [119, 120, 121].

Poliovirus is an icosahedral virus that contains single‐stranded RNA (ssRNA) and is not enveloped. PVSRIPO, a genetically altered version of the Sabin type 1 poliovirus, is the second oncolytic virus to receive Food and Drug Administration (FDA) breakthrough therapy status for recurrent GBM [122]. The natural target of PVS is the poliovirus receptor CD155, which is found in APCs and is increased in GBM. The ability of the virus to infect the nervous system is connected to its internal ribosome entry site, which is exchanged in the engineered version with that of human rhinovirus type 2 [123, 124, 125, 126].

Encased RNA viruses are known as retroviruses. The gamma‐retroviral vector Toca 511 can replicate itself and carries a yeast cytosine deaminase (CD) gene. This gene catalyzes the transformation of 5‐flucytosine (5‐FC), an antifungal medication, into 5‐fluorouracil (5‐FU) that triggers a local antitumor response [127]. In preclinical glioma models, Toca 511 has shown significant oncolytic efficacy [127, 128, 129]. Additionally, it has been demonstrated that Toca 511 at high local concentrations of 5‐FU depletes immunosuppressive myeloid cells in the TME, triggering the development of a T cell‐mediated antitumor immune response [130, 131]. Another clinical trial was conducted using H‐1PV, a DNA virus that infects rats, as a treatment for GBM. The reason why cancer cells are susceptible to H1PV infection is their high levels of essential components for viral replication within the cell, although H‐1PV does not pose any danger to humans [132].

The measles virus (MV), which belongs to the Paramyxovirus family and is a negative single‐stranded RNA virus, has been shown to exhibit oncolytic properties in numerous types of cancer. Naturally occurring oncolytic, weakened vaccine strains of MV have been altered to enhance their specificity for tumors and allow for tracking within living organisms [133, 134]. The MV Edmonton strain (MVEdm) utilizes the carcinoembryonic antigen (CEA) as a reporter gene to monitor viral activity within the body [135]. Glioma animal models treated intratumorally with MV‐CEA showed noticeable tumor shrinkage [136, 137].

Another Oncolytic vaccine virus belongs to the Poxviridae family of enveloped double‐stranded DNA viruses. By incorporating the suicide gene (FCU1) to enhance tumor specificity, TG6002 is a modified Oncolytic vaccine virus with mutations in the ribonucleotide reductase and thymidine kinase genes [138]. In preclinical studies, TG6002 has shown effectiveness in fighting cancer and is currently being tested with 5‐FC in a phase I/II trial involving IV administration of the virus in 78 GBM patients (NCT03294486) [138, 139].

Human cytomegalovirus (CMV) nucleic acids and proteins are initially detected in GBM tissues in over 90% of patients, but not in the normal brain around the tumor [140, 141].

This discovery led researchers to create a peptide immunization against this virus. The vaccination aims to focus on pp6537, the main structural protein of CMV. Research has been done on two methods of treatment: utilizing pp6537‐stimulated DCs and cultivating CMV‐specific CD8^+^ T cells (Table 1) [142, 143, 144]. The study conducted by Reap et al. provides compelling evidence that dendritic cell vaccination (DCV) can enhance the functionality of adoptively transferred CMV‐specific T cells. These T cells exhibit increased polyfunctionality, characterized by concurrent expression of multiple effector molecules such as IFN‐γ, TNF‐α, and IL‐2, suggesting improved antitumor efficacy. The finding underscores the therapeutic potential of CMV‐targeted immunization strategies in GBM, as they have been shown to potentiate T cell polyfunctionality and cytotoxic activity. These approaches may mitigate the profound immunosuppression of the GBM tumor microenvironment, thereby enhancing the efficacy of adoptive and vaccine‐based immunotherapies. By augmenting polyfunctional T cell responses, these approaches offer a promising avenue to strengthen antitumor immunity in this highly immunoevasive malignancy [145]. In addition, complementary evidence from the IMMU‐04 clinical trial reinforces the therapeutic promise of CMV‐targeted vaccination in glioblastoma. In this investigation, nGBM patients were administered a novel CMV peptide‐based vaccine in conjunction with the standard‐of‐care chemotherapeutic agent TMZ, demonstrating the feasibility and potential synergistic efficacy of this combinatorial approach. The results demonstrated the feasibility and safety of this combinatorial approach, and early data indicated the induction of robust CMV‐specific immune responses. Notably, the use of CMV antigens, which are selectively expressed in GBM tissues but not in surrounding normal brain, offers a tumor‐selective target that minimizes off‐tumor toxicity. These outcomes highlight the promise of CMV‐directed immunotherapies and warrant further investigation in larger, controlled trials to validate clinical efficacy and inform optimization of vaccine design [146].

In another major study, Zadeh et al. found that oncolytic virotherapy combined with ICPIs represents a novel strategy for GBM. This study evaluated DNX‐2401, a replicative oncolytic adenovirus, with pembrolizumab in recurrent GBM. Patients received DNX‐2401 followed by pembrolizumab. Tumor responses and immune profiles were monitored. The findings revealed that the combination was well‐tolerated, with partial responses observed in a subset of patients [147]. A phase I/II trial evaluated the reliability of G47∆, a triple‐mutated oncolytic HSV‐1, in patients with progressing GBM after radiation and TMZ treatments. Thirteen patients completed treatment, with 12 experiencing adverse events. The secondary endpoint was efficacy, with a mOS of 7.3 months and a 1‐year survival rate of 38.5%. Median progression‐free survival was 8 days, mostly attributable to the rapid expansion of the contrast‐enhanced region of the target lesion on MRI. Three patients endured for over 46 months, and one entire response was observed at 2 years. Biopsies indicated postadministration MRI characteristics probably represented tumor cell death by viral multiplication and lymphocyte infiltration towards tumor cells. The mechanism of immunological progression is indicative of this treatment. This research shows that G47Δ is suitable for treating recurrent GBM [148]. This collection of studies highlights advancements in immunotherapy for GBM, exploring vaccines targeting specific tumor antigens, such as mutant IDH1, EGFRvIII, and survivin.

Approaches include peptide vaccines such as rindopepimut, SurVaxM, autologous dendritic cell therapies, and oncolytic virotherapy (DNX‐2401) combined with ICPIs. While trials such as ACT IV and Audencel showed limited impact on survival, others demonstrated immune activation and potential clinical benefit, particularly in personalized or combination therapies. These findings underscore the promise of immunotherapy in GBM treatment while emphasizing the need for improved strategies to overcome immune resistance and enhance therapeutic efficacy. Also, an investigation revealed that another cause of GBM may be a mutation in IDH1, so it is a promising target for immunotherapy. A clinical trial was executed to assess the immunogenicity and preliminary efficacy of the IDH1 peptide vaccine. Patients with IDH1‐mutant gliomas were registered and obtained the vaccine with standard therapies. Immune responses were evaluated using T‐cell assays, and clinical outcomes were monitored. The results of this study indicate that the vaccine was well‐tolerated and elicited strong, mutation‐specific T‐cell responses. Early efficacy signals included stabilization of disease in several patients, supporting the potential of this approach for IDH1‐mutant gliomas [70].

#### 2.2.2 Viral Vector Vaccines

Viruses are used as carriers of therapeutic genes to improve cell function or target abnormal cells, with retroviral replicating vectors (RRVs) and Adv being the most studied for GBM [112, 149]. Recombinant viruses, as opposed to oncolytic viruses which kill tumor cells, are designed to safely transport antigens. Without the use of extra adjuvants, these vectors can infect target cells and create antigen peptides, inducing a potent immune response. Viral vectors can strengthen the immune system by utilizing pathogen‐associated molecular patterns (PAMPs); however, prolonged usage may develop antiviral immunity [150, 151]. For example, for the treatment of rodent GBM models, researchers have created a bicistronic Adv vector containing HSV‐1 and a Tet‐inducible expression cassette for Flt3 ligand. The vector has therapeutic efficacy, cytotoxic and immunostimulatory effects, and no excess viral particle burden after injection [152]. There is an ongoing phase I clinical trial that combines immune‐mediated killing triggered by the Flt3L gene with direct destruction of tumor cells. Adv‐tk plus an antiherpetic prodrug are the two drugs used in gene‐mediated cytotoxic immunotherapy (GMCI), and it has demonstrated favorable therapeutic effects on tumor cells [153]. Clinical trials on newly diagnosed, recurring malignant and juvenile malignant gliomas have shown survival advantages and low toxicity [154, 155, 156].

Moreover, powerful vehicles have been engineered using AAV, a viral vector intimately connected to the immune system. To improve T cell function, Ye et al. created a hybrid CRISPR screening system that can target and modify membrane proteins on primary murine T cells in vivo [157]. A vector with VEGF‐C was developed to enhance CD8^+^ T cell activation in mice models of GBM. This vector could enhance antigen removal and change the tumor microenvironment, potentially enhancing the efficacy of immune checkpoint inhibitors (ICPIs) in GBM therapy [158]. In addition to VEGF‐C, GBM frequently displays changes to the epidermal growth factor receptor (EGFR), suggesting that this receptor is essential for the development and proliferation of glial tumors. The RNA polymerase III‐dependent H1 promoter was incorporated into HSV‐1‐based amplicons to allow for the expression of double‐stranded hairpin RNA against EGFR at two distinct sites (pHSVsiEGFR I and pHSVsiEGFR II). Human GBM (gli36‐luc) cell growth was inhibited both in vitro and in vivo by this dose‐dependent posttranscriptional gene silencing using vector‐mediated RNA interference [159]. These findings indicate that effective posttranscriptional gene silencing can be achieved with HSV‐1 amplicons.

Furthermore, RRVs that encode the CD gene can selectively transfect neoplastic cells and convert the prodrug 5‐FC into the cytotoxic agent 5‐FU [160]. Mitchell et al. conducted a study examining the immunogenic properties of RRVs in murine glioma models. Researchers revealed that Toca 511 in conjunction with 5‐FC can elicit a moderated and escalating immune activation. Post‐treatment observations indicated an increase in the expression levels of 41BB, CD40L, and PD‐1, while there was a reduction in the population of immunosuppressive cells. Mice that achieved complete tumor eradication experienced extended survival durations [131].

Recombinant parvoviruses have been utilized to influence the immune response within GBM tumors. In a syngeneic murine model, the transduction of CXCL10 and TNF‐alpha cytokines resulted in tumor regression. This synergistic interaction contributed to a postponement of tumor proliferation in naïve, preestablished tumors; however, no regression was noted in naïve tumors [161]. Notwithstanding the demonstration of significant therapeutic efficacy in clinical trials, viral‐vector gene therapy has yet to attain approval from the FDA. Further progress is required due to limited effectiveness, viral vector delivery, tumor penetration, and safety issues. Even with these challenges, the bright future of viral‐vector gene therapies is supported by the many creative solutions being explored in academia, biotechnology, pharmaceuticals, and manufacturing industries [118].

In addition to these approaches, several viral vector‐based strategies have been developed to deliver immunomodulatory genes directly into the tumor microenvironment. These include inducible IL‐12 expression systems using the RheoSwitch Therapeutic System (RTS), allowing controlled immune stimulation and minimizing systemic toxicity [162]. In this regard, Rivera‐Molina et al. engineered a GITRL‐armed Delta‐24‐RGD oncolytic adenovirus, which extended survival and induced antiglioma immune memory in preclinical models [163]. Passaro et al. constructed an oncolytic HSV‐1 vector encoding a PD‐1‐blocking scFv antibody, providing local checkpoint blockade and improved immune infiltration [164]. In another study, King et al. demonstrated that codelivery of Flt3L and thymidine kinase (TK) via gene therapy led to eradication of multifocal glioma in syngeneic models through combined immunostimulation and direct cytotoxicity [165]. These strategies demonstrate the expanding versatility of viral vectors in not only delivering tumor‐targeting genes but also enhancing antitumor immune responses in glioma therapy.

### 2.3 Cell‐Based Vaccines

#### 2.3.1 Dendritic Cell Vaccination

One particular subset of APCs that regulates immunity and immunological tolerance is the DC. They are considered an interesting goal for eliciting immune responses towards malignancy because they are present in most tissues as immature (resting) cells. They exhibit mature peptides on their human leukocyte antigen (HLA) class I and II receptors after antigen collection and processing, resulting in MHC–peptide complexes. Mature activated DCs move from peripheral tissues to secondary lymphoid organs and lymph nodes in order to engage in physical interactions and trigger T‐cell responses [166].

There is much controversy regarding DCV as a therapeutic adjuvant in GBM. Hundreds of GBM patients have received vaccinations to induce an anticancer immune response through the use of DCV as active immunotherapy in numerous clinical trials. Effectiveness of DCV in GBM generally varies, from no clinical response to notable responses [167]. DCVs increase tumor‐specific IFN‐γ, activate CTL, slow the growth of tumors, and extend life expectancy. The vaccination seems to be safe, well‐tolerated, and free of major side effects (≥ grade 3) [168, 169]. Bota et al. A phase 2 trial evaluated the survival, adverse events, and efficacy of the Aivita GBM vaccine (AV‐GBM)‐1, produced by incubating autologous DCs with irradiated autologous tumor‐initiating cells, using autologous DCs. They determined the treatment was well tolerated; however, there were several treatment‐emergent CNS adverse events (AEs). AV‐GBM‐1 was reliably produced. Treatment was well tolerated. The median Progression‐Free Survival (mPFS) was longer than historical benchmarks, but no mOS improvement was observed [170]. In another study, researchers investigated the efficacy of the autologous tumor lysate‐loaded Dc vaccine (DCVax‐L) in the survival of GBM patients. In this investigation, compared to matched, contemporaneous external controls, adding DCVax‐L to SOC has been connected to a clinically relevant and statistically significant improvement in mOS for patients with both nGBM and recurrent GBM (Table 1) [171].

A phase III trial examined patients who received autologous DC vaccines tailored to tumor antigens alongside standard care. Researchers assessed the survival and immune responses. While overall results were mixed, patients with robust immune responses showed improved survival, underscoring the potential of individualized vaccines [172]. Combining dendritic cell vaccines with radiochemotherapy may enhance immune responses in GBM. Inogés et al. in this phase II trial were evaluated immune activation and survival outcomes. For this reason, patients underwent fluorescence‐guided surgery followed by radio chemotherapy and autologous DC vaccination. The most striking result to emerge from the data is that combination therapy was safe and led to enhanced immune responses. Modest survival benefits were observed, highlighting the potential of integrating DC vaccines into standard GBM treatment [173]. In another study, Audencel, a DC‐based vaccine, was evaluated for its impact on GBM outcomes in a phase II trial. The nGBM patients received Audencel alongside standard therapy. OS and PFS were the primary endpoints. The vaccine showed no significant impact on survival outcomes; therefore, the addition of Audencel to the standard of care did not improve the clinical outcomes of patients with primary GBM [174, 175, 176].

#### 2.3.2 Whole Tumor Cell Vaccines

TSAs and TAAs, which are categorized into five kinds based on their expression in tumors, are used by the immune system to identify cancers [177]. The main goals of the research are to determine which TAA epitopes are the most immunogenic in humans, to characterize the immunogenicity of TAAs, and to investigate their potential as tumor‐defense antigens. A promising strategy to induce a potent antitumor response and long‐term memory is to create vaccines from entire tumor cells, including numerous TAAs recognized by CD8^+^ cytotoxic T lymphocytes (CTLs) and CD4^+^ T helper cells. For efficient tumor regression, this tactic seeks to stimulate the innate and adaptive immune systems [178].

By targeting various tumor antigens and removing the individual epitopes, whole tumor vaccination therapy presents a promising approach to cancer treatment that can be used for all patients, independent of their HLA type [179]. Allogeneic vaccinations offer a more standardized and scalable approach employing tumor cell lines from diverse tumors, whereas autologous vaccines using the tumor cells of patients give customized treatment choices with distinct neo‐tumor antigens. Allogeneic vaccines provide advantages in terms of scalability and quality control, while autologous vaccines have drawbacks regarding reproducibility and tailored therapy [179, 180]. All things considered, whole tumor vaccination therapy offers promise for efficient and customized cancer immunotherapy [181, 182, 183].

### 2.4 Genetic Vaccines for GBM

The fastest‐growing field of vaccine technology is genetic vaccinations, known as gene‐based. In this vaccine technology, cells take nucleic acids like DNA [as plasmids] or RNA [as mRNA] and convert them into proteins in line with the nucleic acid template. Following this protocol may trigger an immune response specific to the tumor [184, 185]. Previous investigations revealed that genetic vaccines such as live or attenuated viruses remarkably trigger MHC class I and class II pathways, enabling the activation of CD8^+^ and CD4^+^ T cells without the inherent danger of live vaccinations [184, 186]. Furthermore, many of the problems associated with recombinant protein‐based vaccinations, including excessive manufacturing costs, challenges with purification, incorrect antigen folding, and inadequate CD8^+^ T cell activation, can be avoided using genetic vaccines [186].

#### 2.4.1 DNA Vaccine

A novel approach being studied in GBM patients is the production of DNA vaccines. Viral origins were highlighted in earlier research as a biological vector for introducing predetermined antigens into host cells. The emphasis has now switched to plasmids made of synthetic DNA [187]. DNA vaccines are perfect for developing cancer vaccines because of a number of their features. Plasmid vaccines pose fewer risks regarding immunogenicity and replication competency than other delivery systems (recombinant proteins and viral vectors). Additional benefits include improved compatibility with people, stability, ease of large‐scale production, and lack of infectious agents [188]. Patients with recently confirmed IDH1 or IDH2 mutations in GBM are participating in a phase I trial of a DNA vaccination. To treat patients with this mutation, this trial makes use of two DNA plasmids: one encoding the tumor‐specific antigen GNOS‐PV01 and the other containing a synthetic DNA plasmid generating the pro‐inflammatory cytokine interleukin‐12, or INO‐9012. Through the promotion of the development of specialized T cells against antigens particular to a patient, IL‐12 functions as a molecular adjuvant to activate the immune system [189]. mRNA vaccine has been described in the following sections.

## 3 mRNA Vaccine for Cancer: The State of Art and the Mechanism of Action

mRNA vaccines are short RNA fragments that are delivered to the body using various methods, including viral vectors like lentiviruses, alphaviruses, and rhabdoviruses, or by encapsulating mRNA within lipid nanoparticles (LNPs) for direct delivery. These mRNA fragments encode specific antigens that, once inside the body, initiate an immune response aimed at identifying and eliminating cancer cells or viral pathogens.

The development of mRNA vaccines for GBM focused on identifying TAAs and TSAs [190, 191], particularly neoantigens arising from somatic mutations that the immune system recognizes as foreign, thereby eliciting potent antitumor T cell responses while avoiding autoimmune reactions [192, 193, 194]. These antigens are detected via assessments of gene expression and genomic changes, such as overexpression [195], mutation frequency [196], and copy number alterations [197]. Moreover, these antigens are selected based on their links to poor prognosis [198, 199] and their association with heightened infiltration of antigen‐presenting cells (APCs) like dendritic cells and macrophages [196, 199]. By delivering these antigens into the body, mRNA vaccines enable APCs to process and display them through MHC class I and II pathways [24], activating CD8+ cytotoxic T cells [200] and CD4+ helper T cells [24]. Dendritic cells are crucial in bridging innate and adaptive immunity [201] by capturing, processing, and presenting antigens, including cross‐presentation of extracellular tumor antigens [202, 203, 204]. This process generates tumor‐specific T cells and triggers pro‐inflammatory cytokines that bolster CD8+ T cell activity, such as Granzyme B, IL‐2, IL‐7, IL‐12, and IL‐15 secretion, leading to better clinical results [205, 206, 207]. Personalized mRNA vaccines are developed by sequencing individual tumor samples to identify unique neoantigens, with antigens showing high expression emerging as promising candidates for effective GBM immunotherapy [198, 208].

After delivery, DCs in the body play a critical role in processing the mRNA. DCs internalize the mRNA fragments through endocytosis or phagocytosis, where ribosomes subsequently decode the mRNA to produce the encoded antigens [209, 210]. These antigens represent parts of the cancer cell or viral structures, which the immune system would not normally recognize as host cells. The antigens produced from mRNA translation are further broken down by proteasomes into smaller peptides. These peptide fragments are then bound by major histocompatibility complex (MHC) molecules within the DCs and presented on their cell surface. This MHC‐antigen complex is essential to activate DCs and prepare them for interaction with other immune cells [211].

Once activated, DCs migrate to the lymph nodes, where they present the antigens to T cells and B cells. In this phase, antigen‐presenting DCs engage with T‐helper cells [CD4^+^ cells] to stimulate the adaptive immune response. Activated T cells, in turn, initiate the production of antibodies by B cells. These antibodies specifically target the antigens displayed by cancer cells or viral pathogens, marking them for destruction [209]. In addition to antibody production, CTLs are activated, especially CD8^+^ cells. These CTLs recognize and directly bind to cancer cells or infected cells displaying the foreign antigens, inducing apoptosis in these targeted cells. This action not only helps eliminate cancer cells but also generates immune memory, reducing the risk of cancer recurrence [211].

**FIGURE 2:**
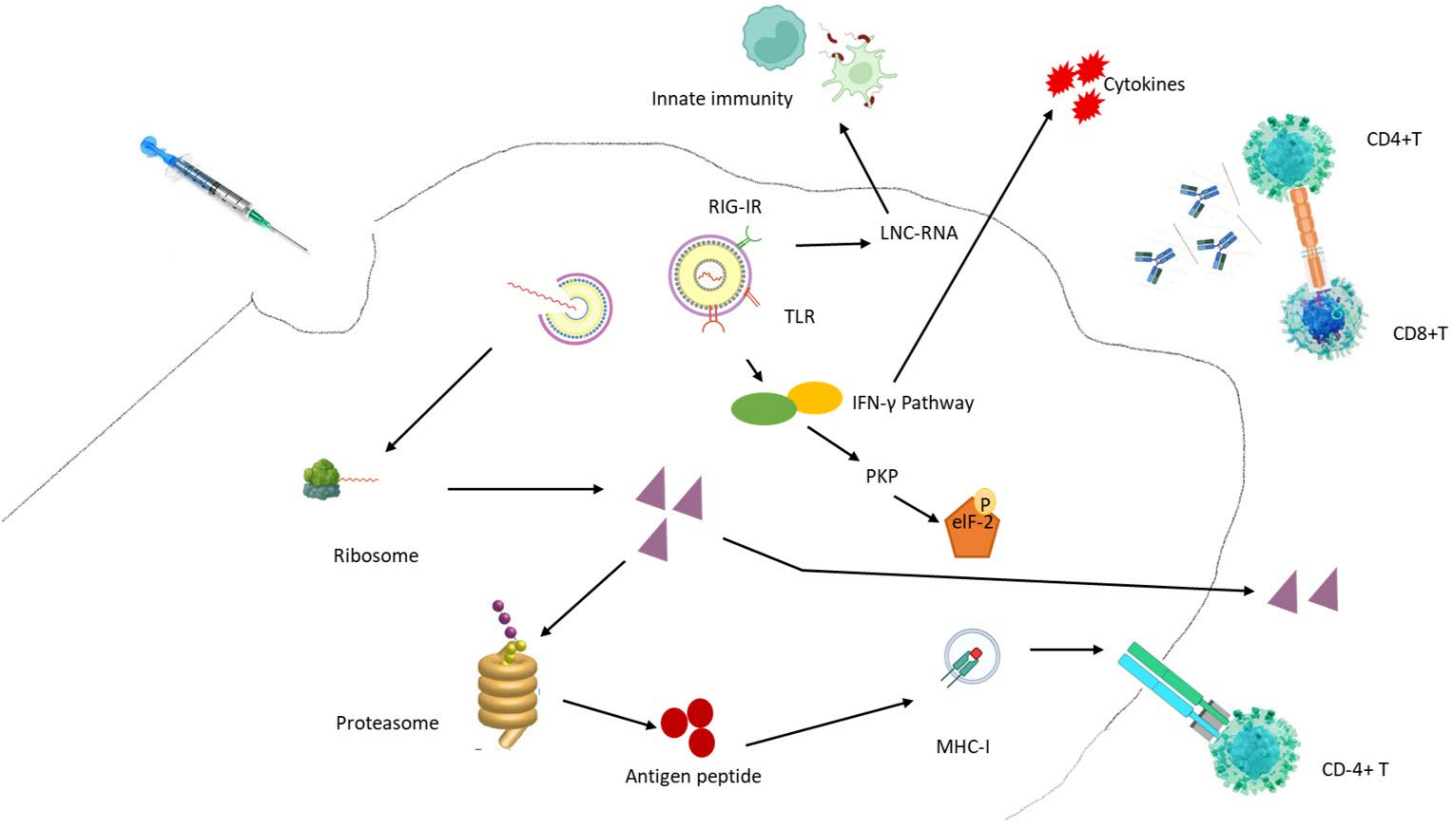
A summary of mRNA vaccine immunity performance. eIF‐2, eukaryotic initiation factor 2; IFN‐γ, interferon; LNC‐RNA, long noncoding RNA; MHC‐I, major histocompatibility complex; PKP, protein kinases; RIG‐IR, retinoic acid‐inducible gene I receptor; TLR, toll‐like receptor 7.

Based on the above, mRNA vaccines stimulate both innate and adaptive immunity. However, before the activation of adaptive immunity, it is crucial to comprehend how cells detect non‐self mRNA and trigger signaling cascades via the interplay of mRNA, pattern recognition receptors (PRRs), and pathogen‐associated molecular patterns (PAMPs). PRRs detect PAMPs either extracellularly via cell surface/endosomal TLRs or intracellularly via RLRs/NLRs [212]. The identification of RNA inside the endosome is mediated by toll‐like receptors (TLRs). Consolidated data indicate that the TLR‐MyD88‐NFκB signaling pathway is often implicated in PAMP identification [213]. TLR‐3 identifies and attaches to double‐stranded RNA (dsRNA), influencing the activation of the type I interferon (IFN) pathway and the release of cytokines and chemokines [214]. Alternatively, ssRNA functions as a PAMP by interacting with TLR‐7 to activate nitric oxide synthase [215]. Cytosolic non‐self RNA is identified by retinoic acid‐inducible gene I (RIG‐I) receptors, nucleotide oligomerization domain‐like receptors, RNA‐dependent protein kinase receptors, and oligoadenylate synthetase receptors. Activated RIG‐I identifies a long non‐coding RNA in conjunction with TRIM25, an E3 ubiquitin ligase that facilitates K63‐linked ubiquitination of RIG‐I, to enhance RIG‐I‐mediated antiviral innate immunity [216]. Another RNA sensor, protein kinase receptor, modulates the transcription factor IRF1, inhibiting the cessation of the translational process to combat the virus [217]. Regardless of the nature of the RNA sensor, RNA‐induced PRRs facilitate the generation of type I IFNs. IFN‐γ augments the production of PKP and the subsequent phosphorylation of eIF2α. Simultaneously, a negative feedback loop is established to inhibit the production of IFN‐γ, impacting mRNA translation and posttranslational modifications [218]. Furthermore, the overexpression of IFN enhances the interaction of oligoadenylate synthetase and dsRNA, facilitating the production of RNase L to destroy non‐self RNA. Consequently, the optimized mRNA vaccines must provide complete activation of innate immunity to induce adaptive immunity. mRNA sequence designers must refrain from excessive activation of innate immunity that obstructs mRNA translation (Figure 2) [219]. In view of all that has been mentioned so far, these mechanisms highlight the innovative design of mRNA vaccines, which employ their own cellular machinery and immune processes to recognize and combat cancer.

### 3.1 Transmission Mechanism

#### 3.1.1 In Vivo and Ex Vivo

The delivery of mRNA vaccines can occur via two general approaches, including in vivo and ex vivo. Both approaches have unique advantages and are chosen based on the specific type of cancer and the desired immune response [209, 210]. In vivo involves direct administration of the mRNA vaccine into the patient, where it is taken up by immune cells such as DCs directly within the tissues. Typically, mRNA is encapsulated in LNPs to protect it from degradation and facilitate efficient cellular uptake [220]. So, LNPs are typically designed to have a size and surface charge that promote uptake by APCs, including DCs, through endocytosis or micropinocytosis [221]. Also, after intramuscular or subcutaneous injection, LNPs drain into lymph nodes, where DCs are highly concentrated. Subsequently, the LNPs enable the mRNA to enter cells and subsequently translate into antigens, triggering an immune response. In vivo delivery is advantageous because it is relatively straightforward and can lead to a rapid, systemic immune response [210].

In contrast, the ex vivo approach involves isolating specific immune cells, often DCs, from the patient and modifying them outside the body. In this process, patient‐derived DCs are extracted and transfected with mRNA in a controlled laboratory setting. Once the DCs have successfully processed the mRNA and presented the desired antigens, they are reintroduced into the patient [222, 223]. This approach offers greater control over the activation of immune cells and is particularly useful for personalized therapies where immune responses must be tightly regulated [224]. Each approach has its benefits. In vivo delivery is less invasive, and it is suitable for broader applications. Despite this, ex vivo delivery allows for a highly customized immune response tailored to individual patients, which is especially advantageous in cancer immunotherapy [225].

#### 3.1.2 mRNA Delivery Systems to Cells in the Vaccination

A key challenge in mRNA vaccine therapy is effective delivery systems. RNA vaccination encounters hurdles such as overcoming barriers that inhibit the entry of foreign nucleic acids and breakdown by RNases. Additionally, the large size of RNA hinders its diffusion within cells, increasing its detection and destruction by the host [226, 227]. Therefore, proper delivery systems are essential [227]. Traditional methods such as in vitro‐loaded DCs, polymer delivery, and mechanical techniques [gene gun, electro injection] are complex, expensive, or unsuitable for humans. Viral vectors are efficient for nucleic acid delivery but face issues such as immunogenicity and manufacturing challenges [228, 229]. Nonviral vectors, while less efficient, are less safe and can carry larger genetic loads, making them easier to synthesize [230, 231]. LNPs are the most common method of non‐viral mRNA delivery [232]. These sub‐micrometer solid structures form an emulsion with solid lipids. LNPs usually have a hydrophilic core and a lipid bilayer shell with different lipids that play different roles. Most formulations use cationic lipids to effectively complex with negatively charged RNA, while anionic and neutral types have also been used. In fact, this electrical charge difference helps to retain the mRNA in the nanoparticles [233, 234]. Novel lipidoids with amine groups maintain a low or neutral surface charge at physiological pH, reduce nonspecific interactions, and facilitate oligonucleotide release into the cytosol. In acidic endosomes, amine groups are ionized, forming a hexagonal phase that disrupts the endosomal membrane and facilitates the escape of mRNA into the cytoplasm (Table 2) [235, 236].

**TABLE 2:** Active or completed clinical trials for mRNA vaccines in glioblastoma.

Vaccine	Trial title	Phase	*N*	Interventions/Treatment	Status	Results	Clinical trial
mRNA vaccines	Vaccine therapy for the treatment of newly diagnosed glioblastoma multiforme (ATTAC‐II)	2	175	pp65‐shLAMP mRNA loaded DC with GM‐CSF and Td combined with stronger doses of TMZ	Completed		NCT02465268
	Viral therapy in treating patients with recurrent glioblastoma multiforme	1	23	Carcinoembryonic antigen‐expressing measles virus	Completed		NCT00390299
	Dendritic cell immunotherapy against cancer stem cells in glioblastoma patients receiving standard therapy (DEN‐STEM)	2/3	60	BTSCs mRNA‐loaded DCs vaccine concomitant with TMZ	Recruiting		NCT03548571
	Safety and tolerability of CVGBM in adults with newly diagnosed MGMT‐unmethylated glioblastoma or astrocytoma	1	16	CV09050101 mRNA vaccine (CVGBM)	Recruiting		NCT05938387
	Vaccine therapy in treating patients undergoing surgery for recurrent glioblastoma multiforme	1	50	BTSC mRNA‐loaded DCs Other study ID numbers	Completed		NCT00890032
DNA vaccines	Neoantigen‐based personalized DNA vaccine in patients with newly diagnosed, unmethylated glioblastoma	1	9	Personalized neoantigen DNA vaccine supplied by Geneos Therapeutics + plasmid encoded IL‐12	Active, not recruiting		NCT04015700
	VXM01 plus avelumab combination study in progressive glioblastoma	1/2	30	Investigational VEGFR‐2 DNA vaccine (VXM01) + Avelumab	Active, not recruiting		NCT03750071
Other types of Genetic vaccines	RNA‐lipid particle (RNA‐LP) vaccines for recurrent adult glioblastoma (GBM)	1	24	pp65 RNA‐LPs vaccine before and after biopsy	Not yet recruiting		NCT06389591

### 3.2 mRNA Vaccines in Glioma

Several studies have found suitable glioma antigens for mRNA vaccine treatment by use of data collected from The Chinese Glioma Genome Atlas (CGGA) and The Cancer Genome Atlas (TCGA), along with TIMER and cBioPortal. Subsequently, they confirm gene modifications and identify immune‐active subtypes. Overall, more than 50 potential antigens have been found for mRNA vaccine development (Table 3). Variations in antigens identified are attributed to the employment of diverse tools and datasets and the complexity of the TME [198].

**TABLE 3:** Studies were conducted to identify appropriate tumor antigens for mRNA vaccination.

Tumor type	Identified antigens	References
Glioma	Isocitrate dehydrogenase 1 (IDH1), transcription factor 12 (TCF12), protein p53 (TP53), complement component 3 (C3)	[237]
Glioma	FKBP prolyl isomerase 10 (FKBP10), glycogen phosphorylase L (PYGL), annexin A5 (ANXA5), moesin (MSN)	[238]
LGG	Filamin C (FLNC), colony‐stimulating factor 2 receptor (CSF2RA), Fc fragment of IgG binding protein (FCGBP), Toll‐like receptor 7 (TLR7)	[239]
Diffuse Glioma	Collagen type I alpha 2 chain (COL1A2), kinase insert domain receptor (KDR), sterile alpha modif domain containing 9 (SAMD9)	[240]
Glioblastoma	Hexokinase 3 (HK3), Actin‐related protein 2/3 complex subunit 1B (ARPC1B)	[241]
Glioblastoma	Cytochrome b‐245 light chain (CYBA), RELT‐like protein 1 (RELL1), major histocompatibility complex (MHC) class I proteins, EGFR	[242]
Glioma	BRCA2, NR5A2, ZNF90, ZNF813, FRRS1, ERCC6L, GTF2H2C, GRAP, ABCB4, NAT1	[243]
Glioblastoma	COL6A1, CYTH4, SAA2, ADAMTSL4, LILRB2, EGFLAM, ADAMTSL4, MPZL2, CTSL	[198]
Glioblastoma	MAN2B1, PLB1, CLEC7A, ARHGAP30, ARPC1B, ARHGAP9	[199]
LGG	IDO1, HOTAIR, RRM2, KIF20A, NR5A2	[244]

There are various difficulties in using mRNA vaccines to treat GBM, and their use is still in the initial stages, requiring more research and clinical trials to fully understand their therapeutic potential [201]. Significant challenges persist before their clinical implementation [245], stemming from the aggressive nature of GBM and its localization in critical brain regions, alongside inherent tumor characteristics like heterogeneity, a highly immunosuppressive TME [245], and low mutational burden [246]. The complex mix of cell types within GBM tumors poses a challenge for universal vaccine development [245], and the immunosuppressive TME, influenced by elements like MDSCs and TAMs, hinders immune responses [247]. Therapeutic agent distribution to brain tumors is significantly limited by the BBB, which poses a formidable challenge [248]. Furthermore, without an efficient delivery route, the enormous size of mRNA molecules hinders cellular uptake, and they are naturally unstable and prone to fast destruction [227, 248, 249]. The absence of well‐characterized, highly immunogenic antigens that are uniquely produced in GBM and that can trigger a robust immune response is another obstacle [18, 250]. Although it is feasible to target TAAs, doing so runs the risk of inducing autoimmunity [243]. There are additional technical challenges, such as creating effective circRNA that expresses proteins or peptides and attaining repeatable, large‐scale production [251]. Investigations into their effectiveness for precisely targeting tumor cells and generating a strong immune response in GBM are still evolving, underscoring the need for technological innovations and a more comprehensive understanding of immune dynamics specific to GBM [201]. Additional research is essential to confirm and refine their performance, particularly by addressing immune cell exhaustion and refining patient selection methods based on immune profiles [198].

As we mentioned above, BBB poses a significant obstacle, restricting the effective transport of therapeutic agents, such as mRNA vaccines, to brain tumors like GBM [252, 253, 254]. To address this, advanced nanoscale systems are essential for penetrating the BBB, precisely targeting GBM cells, and reducing harm to surrounding healthy tissue [248, 255, 256]. Researchers are investigating various nanoparticle‐based platforms for controlled and site‐specific delivery [257, 258], including LNPs [249], polymeric nanoparticles [259], liposomes [260], exosomes [261], and biomimetic nanoparticles [262]. mRNA can be encapsulated by nanoparticles to prevent deterioration [248, 249] and can be engineered with targeted ligands on their surfaces to enhance BBB traversal and cellular uptake [263]. Notably, biomimetic nanoparticles replicate natural biological structures, like cell membranes, allowing them to avoid immune responses and efficiently breach the BBB [262]. Exploiting the partial breakdown of the BBB in the core of GBM tumors, which boosts permeability [262, 264], presents a viable strategy for mRNA vaccine delivery. Nevertheless, the intact BBB at the tumor's periphery continues to pose a significant barrier [265]. Other tactics involve direct local delivery techniques, such as intracranial or intrathecal administration, to sidestep the BBB entirely [266], as well as leveraging mRNA to stimulate lymphangiogenesis, thereby enhancing immune cell infiltration and antigen transport to peripheral lymph nodes [267, 268]. Despite their potential, these methods remain largely experimental or in early clinical stages, with ongoing hurdles in refining delivery mechanisms and production processes [201].

mRNA vaccines provide several key benefits compared to conventional vaccine approaches, such as strong tolerability, no risk of genomic integration, inability to induce infections, streamlined and economical production, and the capacity to stimulate both cellular and antibody‐mediated immune responses [24]. In contrast to peptide vaccines, which are limited to partial antigens and HLA restrictions, mRNA vaccines can encode complete tumor antigens, enabling a more diverse T‐cell activation [208, 260]. Given the variability in GBM tumors, vaccines targeting a single antigen, as with some peptide options, have yielded modest results [117], whereas mRNA vaccines support the simultaneous delivery of multiple antigens [187]. Cell‐based DC vaccines have demonstrated safety and practicality, with select trials showing extended patient survival [172], and mRNA can be leveraged to effectively load these DCs [201]. Nonetheless, mRNA vaccines are hindered by issues like inherent instability, rapid enzymatic breakdown [260], excessive immune reactivity, and inefficient delivery in the body, which have constrained their clinical adoption [269]. Unlike ICIs and CAR‐T cell therapies, which have demonstrated substantial efficacy in various other cancers [270, 271, 272], GBM introduces specific hurdles, such as a low mutational load, an immunosuppressive tumor microenvironment, and the BBB [246, 273], that often result in limited clinical responses in ICI studies [274]. Although CAR‐T therapies have shown promise against targets like IL13 Rα2 and EGFRvIII, ongoing issues with uneven antigen distribution and antigen escape continue to pose challenges [275, 276, 277]. In contrast, personalized mRNA vaccines targeting neoantigens present a flexible and innovative option [278], capable of countering tumor variability and individual patient differences [279] while potentially activating immune responses in “cold” tumor profiles, such as the GBM IS1 subtype [198]. Continued studies are essential to refine and confirm the role of mRNA vaccines in tackling this aggressive disease.

Ma et al. detected suitable antigens and immune subtypes (IS) for mRNA vaccine development against LGG and GBM. The association between genes and immune cell infiltration, along with confirmation of gene modifications, was established via TIMER and cBioPortal, respectively. This study determined four antigens, including IDH1, transcription factor 12 (TCF12), protein p53 (TP53), and complement component 3 (C3), as being potentially effective for mRNA vaccine development. Additionally, this study categorized glioma into four immune subtypes (IS1–IS4), with each subtype correlating with cellular, molecular, and clinical features. The study found that the IS1 and IS4 subtypes, which have elevated single‐nucleotide polymorphisms (SNPs), single‐nucleotide variants (SNVs), total mutational number, and HLA molecule expression, are more likely to be responsive to mRNA vaccines. These subtypes have immune‐active phenotypes due to higher scores for activated B cells and CD8^+^ T cells, while the IS2 and IS3 subtypes have immune‐suppressive phenotypes due to higher scores for memory B cells and CD4^+^ T cells [237]. Similarly, Zhong et al. conducted a study to identify suitable candidates for glioma mRNA vaccination using RNA sequence and clinical data from CGGA and TCGA. They used cBioPortal for genetic modification profile visualization and TIMER for APC infiltration calculation. Four glioma antigens were identified, including FKBP prolyl isomerase 10 (FKBP10), glycogen phosphorylase L (PYGL), annexin A5 (ANXA5), and moesin (MSN), which were correlated with elevated APC infiltration and better prognoses. The study also identified three ISs comprising IS1, IS2, and IS3. They found that among them, IS2 was a suitable vaccine candidate [238]. Likewise, Ye et al. conducted a study to identify potential LGG tumor antigens and their corresponding immune groups for mRNA vaccination. They used data from TIMER and identified four potential antigens, including filamin C (FLNC), colony‐stimulating factor 2 receptor (CSF2RA), Fc fragment of IgG binding protein (FCGBP), and Toll‐like receptor 7 (TLR7). They also identified three distinct ISs containing desert, immune inhibition, and inflamed. Researchers found that inflamed subtypes were the most suitable for LGG vaccination [280]. Zhou et al. used gene‐expression profiles and clinical data from CGGA and TCGA datasets to identify potential antigens and suitable IS for diffuse glioma vaccination. They identified three potential antigens, including collagen type I alpha 2 chains (COL1A2), kinase insert domain receptor (KDR), and sterile alpha motif domain containing 9 (SAMD9). They also identified three IS (IS1–IS3), with IS1 being an immunologically cold phenomenon with a poorer prognosis and suggesting it as a better target for immunotherapy [240]. In a parallel investigation, Ye et al. performed a study to identify suitable tumor antigens and ISs for GBM. They used genomic and clinical data from the TCGA and TIMER to examine the association between immune cell infiltration and detected antigens. They found hexokinase 3 and actin‐related protein 2/3 complex subunit 1B (ARPC1B) to be highly correlated with APCs in GBM. Immunophenotyping identified two clinically distinct ISs: immune inhibition and immune inflamed [241]. Furthermore, Rose et al. compared the surfaceomes of GBM with astrocyte cell lines to identify potential GBM treatment targets. They used cell surface protein biotinylation, streptavidin beads purification, and shotgun proteomics analysis. They identified 11 potential GBM targets, including five mutated proteins, like cytochrome b‐245 light chain (CYBA), RELT‐like protein 1 (RELL1), MHC class I, and EGFR. Seven of these proteins, including CYBA, MHC class I, EGFR, B‐41 alpha chain (hla‐b), A‐24 alpha chain (hla‐a), prolyl 4‐hydroxylase subunit alpha 2 (P4HA2), carboxypeptidase M (CPM), and HSPD1, are currently targeting in clinical trials [242]. Furthermore, Chen et al. employed the CGGA, TCGA‐LGG, and TCGA‐GBM databanks to identify ten potential antigens for glioma vaccination, including BRCA2, NR5A2, ZNF90, ZNF813, FRRS1, ERCC6L, GTF2H2C, GRAP, ABCB4, and NAT1. They also identified five distinct ISs, with the IS2A/2B subtype within the IS2 recommended for vaccination [243]. Wu et al. investigated a study using RNA sequence, clinical data, and microarray data to identify potential tumor antigens and ISs of GBM patients for personalized vaccine development. The study identified nine potential antigens correlated with depressed APC infiltration and worse prognoses, including COL6A1, CYTH4, SAA2, ADAMTSL4, LILRB2, EGFLAM, ADAMTSL4, MPZL2, and CTSL. The study also identified four distinct IS (IS1–IS4), with IS1 being immunologically inactive, IS3 being cold, IS4 having moderate TME, and IS2 exhibiting hot and immunosuppressive properties. The authors suggested that the IS2 GBM group is more suitable for ICI therapy, while the IS1 group is more suitable for mRNA vaccination [198]. Additionally, Lin et al. identified six mutated tumor antigens using RNA sequence, clinical data, and TIMER, GEPIA, and cBioPortal. They evaluated genetic modifications and altered expression profiles of GBM antigens. These include MAN2B1, PLB1, CLEC7A, ARHGAP30, ARPC1B, and ARHGAP9. They also identified IS1, IS2, and IS3, with immune‐active, intermediate, and suppressive phenotypes, respectively [199]. Also, Zhao et al. developed an mRNA vaccine for LGG by ferroptosis‐linked antigens. They used CGGA and TCGA datasets to obtain genomic and clinical information. Five ferroptosis‐related gene‐based antigens were identified, including IDO1, HOTAIR, RRM2, KIF20A, and NR5A. Upregulation of these genes was associated with poorer OS, progression‐free survival, increased APCs, and B cell infiltration. LGG was classified into four subtypes (FS1–FS4), with FS1 and FS3 being immunologically hot phenotypes, and FS2 and FS4 being immunologically cold phenotypes [244].

The studies on mRNA vaccines for GBM have limitations, including the lack of large‐scale clinical trials to validate their therapeutic efficacy. The small sample size limits patient stratification by age, gender, and pathological classifications, potentially explaining the observed heterogeneity. Selection bias may affect outcomes, and potential confounding factors must be acknowledged. Despite these limitations, the preliminary results of studies are crucial for further research.

## 4 Current Status and Future Prospective

GBM is one of the most aggressive and treatment‐resistant forms of cancer, with limited treatment options and a poor prognosis [281]. Over the years, the development of innovative therapies has been a critical area of research, and mRNA vaccines have emerged as a promising strategy for the treatment of GBM. mRNA vaccines have shown potential in targeting TSAs and enhancing immune responses against cancer cells. The success of mRNA vaccines in combating other cancers, such as melanoma, has provided a foundation for similar approaches for GBM [282, 283]. Research has identified several glioma‐associated antigens, such as IL13Ra2 and EGFRvIII, that can be targeted using mRNA vaccines. These vaccines stimulate the immune system to recognize and destroy tumor cells expressing these antigens. Preclinical models have demonstrated that mRNA vaccines can induce robust antitumor immune responses in GBM [284]. Early‐phase clinical trials, including the testing of personalized mRNA vaccines developed from the tumor's specific genetic mutations, have shown promising results in improving immune response and survival rates in some GBM patients. However, effective delivery systems for mRNA vaccines remain a crucial challenge. LNPs have been widely used as mRNA delivery vehicles and have shown promise in clinical settings [232]. Also, new advancements in lipid nanoparticle formulations and other delivery methods such as electroporation and viral vectors have enhanced the stability and bioavailability of mRNA vaccines, improving their efficacy [285].

However, despite the many advantages, there are still limitations regarding these types of vaccines for GBM. While mRNA vaccines targeting glioma‐associated antigens show promise, many of these antigens are not exclusive to GBM, and their expression is not uniform across all patients. This creates variability in treatment outcomes. Moreover, the immunosuppressive tumor microenvironment in GBM poses a significant barrier to the effectiveness of mRNA vaccines. The presence of myeloid‐derived suppressor cells (MDSCs), regulatory T cells (Tregs), and tumor‐associated macrophages (TAMs) dampens immune responses and limits the efficacy of the vaccine. Additionally, effective delivery of mRNA vaccines to the tumor site remains a major hurdle. Despite advances in lipid nanoparticles, many patients still experience limited uptake and stability of mRNA vaccines, which can impact therapeutic outcomes.

Accordingly, further research is needed to assess their long‐term safety, particularly in the context of a highly aggressive and metastatic cancer like GBM. Because a major knowledge gap lies in the identification of universal or widely applicable tumor antigens that could be targeted across all GBM patients. There is a need for deeper exploration into the genetic and epigenetic profile of GBM to uncover novel TSA. Also, while mRNA vaccines can stimulate the immune system, they may not overcome the immune evasion mechanisms employed by GBM. Additionally, there is growing evidence that combining mRNA vaccines with other treatment modalities, such as ICIPs, oncolytic viruses, or chemotherapy, may enhance their efficacy.

The subsequent studies should concentrate on enhancing vaccine design, improving delivery methods, and identifying novel antigenic targets. Additionally, overcoming the immunosuppressive tumor microenvironment through combination therapies and enhancing the long‐term persistence of immune responses will be crucial for the development of effective vaccines. By addressing these gaps, future studies could pave the way for novel, life‐saving treatments for patients with GBM. One of the most promising avenues for future mRNA vaccine development is the use of personalized cancer vaccines based on individual tumor mutations. Advances in high‐throughput sequencing technologies, such as next‐generation sequencing (NGS), will enable the identification of unique neoantigens that are specific to the patient, ensuring that the mRNA vaccine targets the most relevant TAAs. Also, the future of mRNA vaccines for GBM treatment will depend on collaboration between multiple disciplines, including molecular biology, immunology, bioengineering, and computational biology. Overall, the future of mRNA vaccines for GBM is bright, driven by technological innovation, personalized treatment strategies, and interdisciplinary collaboration. By overcoming current challenges related to antigen specificity, tumor microenvironment immunosuppression, and vaccine delivery, we are poised to see the advent of highly effective, targeted therapies that can dramatically improve the survival and quality of life for GBM patients. With continued advancements in immuno‐oncology, precision medicine, and regulatory policy, mRNA vaccines could become a cornerstone of GBM therapy, marking a new era in the treatment of this aggressive and devastating cancer.

## 5 Conclusion

GBM, a highly aggressive and treatment‐resistant form of cancer, has been the focus of research for years. Current research focuses on identifying and targeting GBM‐specific antigens, such as WT1, survivin, IDH1, EGFRvIII, TERT, and HSPs. These antigens are crucial for stimulating a targeted immune response. mRNA vaccines have surfaced as a possible approach for treating GBM by targeting TSAs and enhancing immune responses against cancer cells. Research has identified several glioma‐associated antigens that can be targeted using mRNA vaccines, stimulating the immune system to recognize and destroy tumor cells expressing these antigens. Preclinical models have illustrated that mRNA vaccines can induce robust antitumor immune responses in GBM. Early‐phase clinical trials, including the testing of personalized mRNA vaccines developed from the tumor's specific genetic mutations, have shown promising results in improving immune response and survival rates in some GBM patients. However, effective delivery systems for mRNA vaccines remain a crucial challenge. LNPs have shown promise in clinical settings, and new advancements in lipid nanoparticle formulations and other delivery methods, such as electroporation and viral vectors, have enhanced the stability and bioavailability of mRNA vaccines. However, there are still limitations regarding these types of vaccines for GBM, including the delivery of vaccines across the BBB, the induction of a strong and sustained immune response, and the ability to overcome tumor immune evasion mechanisms. In conclusion, overcoming current challenges can dramatically enhance survival alongside the standard of life for GBM patients.

## Author Contributions

We declare that we contributed significantly towards the research study that is, (a) conception and design (S.B., S.G., P.H., S.P.) and (b) drafting the article or revising it critically for important intellectual content (H.H., M.J.‐N.) and on (c) final approval of the version to be published (H.H., M.J.‐N.).

## Ethics Statement

The authors have nothing to report.

## Consent

The authors have nothing to report.

## Conflicts of Interest

The authors declare no conflicts of interest.

## Data Availability

Data sharing not applicable to this article as no datasets were generated or analyzed during the current study.
